# Simultaneous Dual-Wavelength Laser Irradiation against Implant-Adherent Biofilms of *Staphylococcus aureus*, *Escherichia coli*, and *Candida albicans* for Improved Antimicrobial Photodynamic Therapy

**DOI:** 10.3390/bioengineering11010048

**Published:** 2024-01-02

**Authors:** Shima Afrasiabi, Stefano Benedicenti, Antonio Signore, Mahnaz Arshad, Nasim Chiniforush

**Affiliations:** 1 Laser Research Center of Dentistry, Dentistry Research Institute, Tehran University of Medical Sciences, Tehran 1441987566, Iran; shafrasiabi@alumnus.tums.ac.ir; 2 Department of Surgical Sciences and Integrated Diagnostics, University of Genoa, Viale Benedetto XV, 6, 16132 Genoa, Italy; benedicenti@unige.it; 3 Therapeutic Dentistry Department, Institute of Dentistry, I.M. Sechenov First Moscow State Medical University, Trubetskaya Str. 8, b. 2, 119992 Moscow, Russia; dr.signore@icloud.com; 4 Dental Research Center, Dentistry Research Institute, Tehran University of Medical Sciences, Tehran 1441987566, Iran; mahnazarshad@yahoo.com; 5 Department of Prosthodontics, School of Dentistry, International Campus, Tehran University of Medical Sciences, Tehran 1441987566, Iran

**Keywords:** antimicrobial photodynamic therapy, dental implants, biofilms, hydrogen peroxide

## Abstract

The efficiency of antimicrobial photodynamic therapy (PDT) might be improved by using multiple wavelengths. This study investigates the sensitivity of implant-adherent biofilms of *Staphylococcus aureus*, *Escherichia coli*, and *Candida albicans* to indocyanine green (ICG)-808 nm diode laser, toluidine blue O (TBO)-635 nm diode laser, and hydrogen peroxide (HP)-980 nm diode laser and their combination when irradiated with dual-wavelength laser irradiation (simultaneously 980–635 nm or 980–808 nm). After an incubation period of 72 h, the infected implants were randomly divided into seven different treatment modalities: Control, HP, HP-PDT, TBO-PDT, HP-TBO-PDT, ICG-PDT, and HP-ICG-PDT. After the treatments, the colony-forming units (CFUs)/mL and reactive oxygen species (ROS) generation were determined. All evaluated disinfection methods were significantly effective against the three investigated bacteria compared to the control. The combined treatment of HP-ICG-PDT or HP-TBO-PDT had the greatest antibacterial effect compared to each treatment alone. There were statistical differences between HP-ICG-PDT and ICG-PDT or HP-TBO-PDT and TBO-PDT for all three bacteria studied. PDT with simultaneous dual-wavelength laser irradiation is an efficient strategy to improve the therapeutic effect of PDT.

## 1. Introduction

Replacing missing teeth with dental implants is a standard permanent treatment today. Nevertheless, pathological conditions can develop in the peri-implant tissues, endangering the implants and their reconstruction and possibly also affecting the health of the patients [1]. Bacterial colonization and biofilm formation begin shortly after implant placement, and growth and maturation continue on the implant and tooth surfaces. The interaction of implant-associated biofilms with host cells can cause peri-implant mucositis, a local inflammatory reaction of the oral mucosa. Although peri-implant mucositis is reversible, it can progress to peri-implantitis, a more severe inflammatory disease characterized by alveolar bone loss [2]. Peri-implantitis is one of the most common complications in dental implantology, with a prevalence of 19.83% and affects both the surrounding soft and hard tissues and can lead to implant loss [3].

Some bacterial species, such as *Aggregatibacter actinomycetemcomitans*, *Porphyromonas gingivalis*, *Tannerella forsythia*, and *Prevotella* spp., are commonly found in peri-implant diseases [4]. Currently, some microorganisms, such as *Staphylococcus aureus*, *Staphylococcus epidermidis*, *Enterobacter aerogenes*, *Enterobacter cloacae*, *Escherichia coli*, *Helicobacter pylori*, *Parvimonas micra*, *Pseudomonas* spp., and *Candida* spp., have been identified around the implant that are not common in periodontitis patients [5]. *S. aureus*, *E. coli*, and *C. albicans* are microorganisms that are frequently involved in peri-implantitis [5,6,7,8]. *C. albicans* may be involved in the initiation and development of peri-implantitis, as *Candida* colonization and biofilm formation on other metal surfaces such as hip and knee prostheses are relatively common [6].

Metal instruments and ultrasonic scalers with or without local antiseptic therapy can increase the surface roughness of implants, and non-metallic curettes do not provide effective removal of the microbial plaque. Chemical agents do not allow complete removal of bacterial contamination and may contaminate the implant surface with different chemical agents [9]. Diode lasers with different wavelengths have been effective in decontaminating implants and inhibiting lipopolysaccharide (LPS)-induced macrophage activation and consequent attenuation of the inflammatory response. They do not alter the physical structure of the implant surface [10,11].

Antimicrobial photodynamic therapy (PDT) is a minimally invasive antimicrobial approach that has been proposed as a complementary treatment for peri-implantitis [12]. PDT consists of three main components: A light source with a specific wavelength, a photosensitizer, and oxygen, which, by activating the photosensitizer in the presence of oxygen, leads to the formation of reactive oxygen radicals that can cause cell death [13]. PDT is a successful method in the treatment of periodontal infections [14]. Light activation enables better efficacy in topical form and reduces the side effects of PDT [15]. Although the efficacy of the PDT method in reducing the microbial load and virulence of bacteria has been demonstrated in laboratory and in vivo studies with minimal invasion and tissue damage, there is still a need to improve the efficiency of this method. Since the type and concentration of the photosensitizer and the light source have an impact on the efficiency of this method to maximize the production of oxygen-free radicals, one can try to improve the efficiency of this method by changing the types and also the formulation [16,17].

Local administration of antiseptics has been proposed as an effective method in nonsurgical treatment for decontamination of the implant surface in the treatment of peri-implantitis, but hydrogen peroxide (HP) has been shown to be toxic to cells when used in high concentrations or with long-term exposure. Therefore, it is recommended to combine the administration of antiseptics with other conservative treatments [18]. The strategy of combining antimicrobial agents with PDT is now of interest as it often brings advantages in biofilm eradication [19]. Deeper penetration into biofilms as well as low interference from radiation are the advantages of HP [20]. The use of HP as a photosensitizer has been investigated in several studies [21,22,23].

Although the concept of photosensitizer-based PDT is widely recognized, most published work to date has focused on traditional PDT. Therefore, there is a research gap in the development of innovative light sources and photosensitizers for improved PDT. This study aims to employ HP and evaluate the antimicrobial effect of simultaneous dual-wavelength laser irradiation together with ICG and TBO on implant-adherent biofilms of *S. aureus*, *E. coli*, and *C. albicans* for improved PDT.

## 2. Materials and Methods

### 2.1. Photosensitizer and Light Source

SIOXYL Solution (3% HP in stabilized water solution glycerophosphate) [SIOXYL Solution, Doctor Smile, Italy], TBO (Merck KGaA, Darmstadt, Germany) at a concentration of 0.1 mg/mL, and ICG (green +I, Novateb Pars, Tehran, Iran) at a concentration of 1 mg/mL were used as photosensitizers in this study. A diode laser (Wiser 3, Doctor Smile, Italy) with 3 wavelengths, including 635 nm, 808 nm, and 980 nm was used. A diode laser with 980 nm at a peak power of 2.5 W (average power of 800 mW) and T-ON 30 μs and T-OFF 70 μs, with a frequency of 10 kHz and an average energy density of 96 J/cm^2^, was used to activate HP alone. For TBO activation, a diode laser at a wavelength of 635 nm with an output power of 200 mW and an energy density of 24 J/cm^2^ in continuous mode was used, and for ICG, a diode laser at a wavelength of 808 nm with an output power of 200 mW and an energy density of 24 J/cm^2^ in continuous mode was used. For the mixed groups, HP-TBO-PDT and HP-ICG-PDT, the parameters of 980–635 nm or 980–808 nm were applied simultaneously with the same parameters as above. The irradiation distance was set to 2 mm. The irradiation time was also adjusted to 60 s, and the tip area was 0.5 cm^2^ for all groups.

### 2.2. Biofilm Formation

The experiments were performed on a total of 105 titanium implants (Dentium, Seoul, Republic of Korea), 10 mm in diameter and 4.1 mm thick. *S. aureus* (IBRC-M10917), *E. coli* (IBRC-M10698), and *C. albicans* (ATCC 10231) were used for biofilm formation on implant surfaces. The microorganisms were prepared in brain heart infusion (BHI) broth (Merck, Darmstadt, Germany) at a concentration of 10^7^ colony-forming units (CFUs)/mL. *C. albicans* was cultured on Sabouraud dextrose (SD) broth (Ibresco, Tehran, Iran). Each implant was then contaminated separately with 1 mL of each bacterial suspension in sterile 12-well microplates. This was followed by incubation for 72 h in a 5% CO_2_ atmosphere at 37 °C.

### 2.3. Treatment Groups

**Figure 1:**
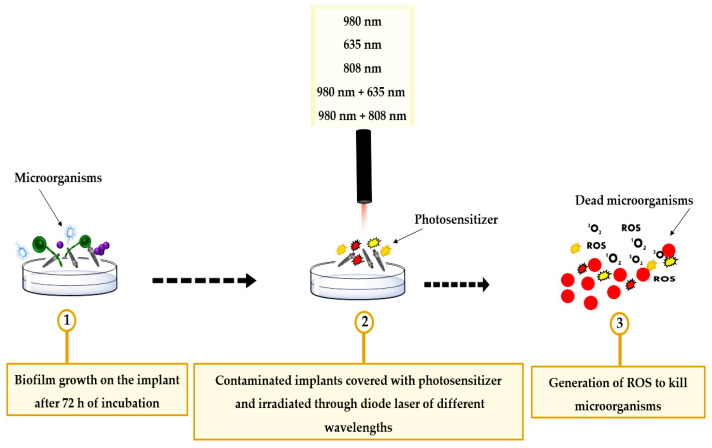
A schematic drawing showing the methodological design of the study groups. ROS: reactive oxygen species.

At the end of the incubation period, the implants are gently washed with phosphate-buffered saline (PBS) and then divided into three groups depending on the bacteria with which they were contaminated: Group 1 (*S. aureus*), Group 2 (*E. coli*), and Group 3 (*C. albicans*). Finally, the 35 titanium implants in each group were randomly divided into seven treatment subgroups (n = 5 per subgroup) as follows (Figure 1):

Treatment 1 (Control): The implants remained untreated.

Treatment 2 (HP): The implants were submerged in 1 mL of HP solution. This was left in the dark for 2 min.

Treatment 3 (HP-PDT): The implants were submerged in 1 mL of HP solution. This was left in the dark for 2 min. Subsequently, the implants were irradiated with a diode laser with a wavelength of 980 nm and an average power of 800 mW for 60 s.

Treatment 4 (TBO-PDT): The implants were submerged in 1 mL of TBO solution at a concentration of 0.1 mg/mL. This was left in the dark for 5 min. Subsequently, the implants were irradiated with a diode laser with a wavelength of 635 nm and an output power of 200 mW for 60 s.

Treatment 5 (HP-TBO-PDT): The implants were submerged in 1 mL of HP solution. This was left in the dark for 2 min. Next, the implants were placed in 1 mL of TBO solution at a concentration of 0.1 mg/mL for 5 min. Subsequently, the implants were irradiated simultaneously with a diode laser with a wavelength of 980 nm–635 nm for 60 s.

Treatment 6 (ICG-PDT): The implants were submerged in 1 mL of ICG solution at a concentration of 1 mg/mL. This was left in the dark for 5 min. Subsequently, the implants were irradiated with a diode laser with a wavelength of 808 nm and an output power of 200 mW for 60 s.

Treatment 7 (HP-ICG-PDT): The implants were submerged in 1 mL of HP solution. This was left in the dark for 2 min. Next, the implants were placed in 1 mL of ICG solution at a concentration of 1 mg/mL for 5 min. Subsequently, the implants were irradiated simultaneously with a diode laser with a wavelength of 980 nm–808 nm for 60 s.

### 2.4. Plate Count Method

After treatment, each Eppendorf tube containing the implant was filled with 1 mL of PBS, and biofilms were detached by vigorously vortexing for 60 s and then resuspended by pipetting up and down. Serial 5-fold dilutions of each sample were made in PBS, and 10 μL of each dilution was plated onto BHI agar (Ibresco, Tehran, Iran) to determine the total cell number of each microorganism. For *C. albicans*, SD agar plates (Ibresco, Tehran, Iran) were used.

### 2.5. Measuring Reactive Oxygen Species (ROS)

The generation of ROS by microorganisms involved in implant-adherent biofilms after treatment with experimental groups was determined using 2′,7′-dichlorofluorescin diacetate (DCFH-DA). The cell suspension was used for measuring ROS formation. To evaluate ROS generation, 10 µL of DCFH-DA stock solution and 162 µL assay buffer were added to 50 µL of samples of the microorganisms and incubated at 37 °C for 15 min. The oxidation of DCFH-DA to DCF at 485 nm excitation and 528 nm emission was measured by an ELISA fluorimeter (Bio Tek, synergy^TM^ HT, Winooski, VT, USA) [24].

### 2.6. Statistical Analysis

Data were analyzed using the statistical software package IBM SPSS Statistics version 26.0 (IBM, Chicago, IL, USA). Mean l g CFU/mL ± standard deviation was demonstrated for each condition. In general, five independent experiments were performed. One-way analysis of variance (ANOVA) tests were applied to evaluate significant differences between groups. A Tukey correction was applied to the *p*-value to account for multiple comparisons of data. Differences were considered statistically significant for *p*-values < 0.05.

## 3. Results

### 3.1. Effects of Treatment Groups on the Cell Viability

**Figure 2:**
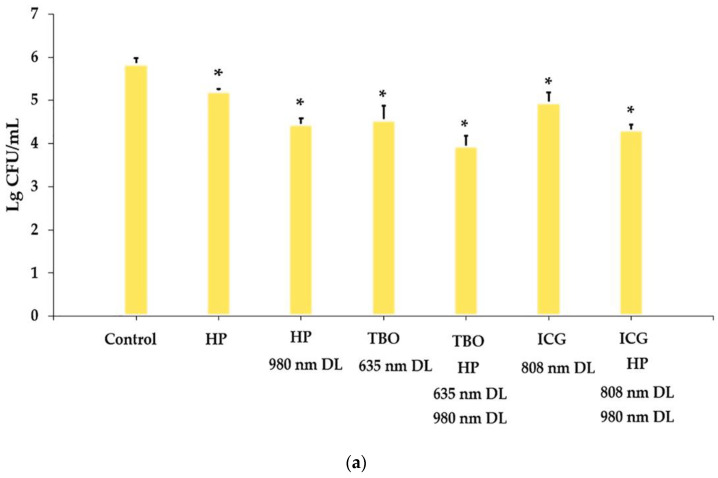

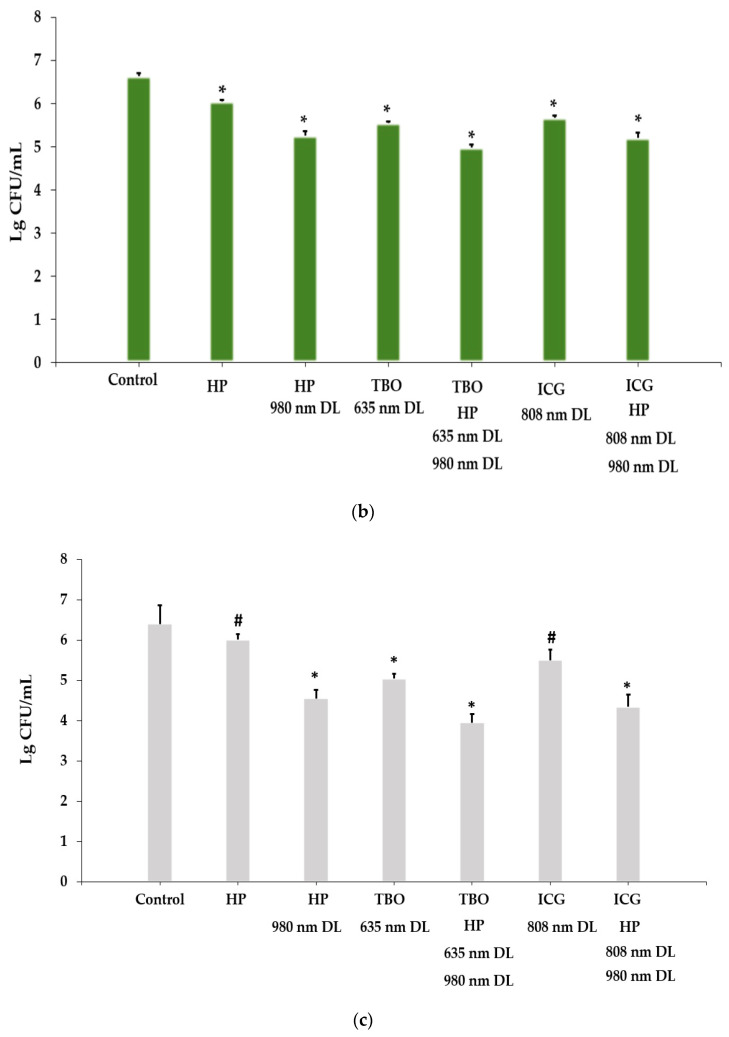
Cell viability of *Staphylococcus aureus* (**a**), *Escherichia coli* (**b**), and *Candida albicans* (**c**) biofilms in an experimental implant-related infection before and after treatment. Values are shown as mean lg CFU/mL ± standard deviation. # = *p* < 0.05, * = *p* ≤ 0.001. HP: hydrogen peroxide; DL: diode laser; TBO: toluidine blue O; ICG: indocyanine green.

For *S. aureus*, statistically significant differences were found between all treatment groups and the control (*p* < 0.001, Figure 2a). The HP-TBO-PDT group showed significant differences from TBO-PDT (*p* = 0.01) and significant differences compared with HP-PDT (*p* = 0.037). This study showed that the HP-ICG-PDT group was significantly more effective than the ICG-PDT (*p* = 0.006), but not compared to the HP-PDT (*p* = 0.915).

For *E. coli*, significant differences were observed for all treatment groups with regard to the control (*p* < 0.001, Figure 2b). The HP-TBO-PDT group showed significant differences compared to TBO-PDT and HP-PDT (*p* < 0.001 and *p* = 0.006, respectively). Treatment with HP-ICG-PDT showed more antibacterial effects than ICG-PDT (*p* < 0.001). There was no significant difference in the bacterial count of *E. coli* between the HP-ICG-PDT group and the HP-PDT group (*p* = 0.968).

In the case of *C. albicans*, the differences compared to the control were significant for all treatment groups (*p* = 0.011 for ICG-PDT and *p* ≤ 0.001 for the others, Figure 2c). The HP-TBO-PDT group showed significant differences compared to TBO-PDT and HP-PDT (*p* = 0.008 and *p* = 0.047, respectively). The CFU/mL in the HP-ICG-PDT group was significantly lower than in the ICG-PDT group but not in the HP-PDT group (*p* = 0.003 and *p* = 0.401, respectively). The details of the analysis are shown in Table 1.

### 3.2. Intracellular ROS Generation

**Figure 3:**
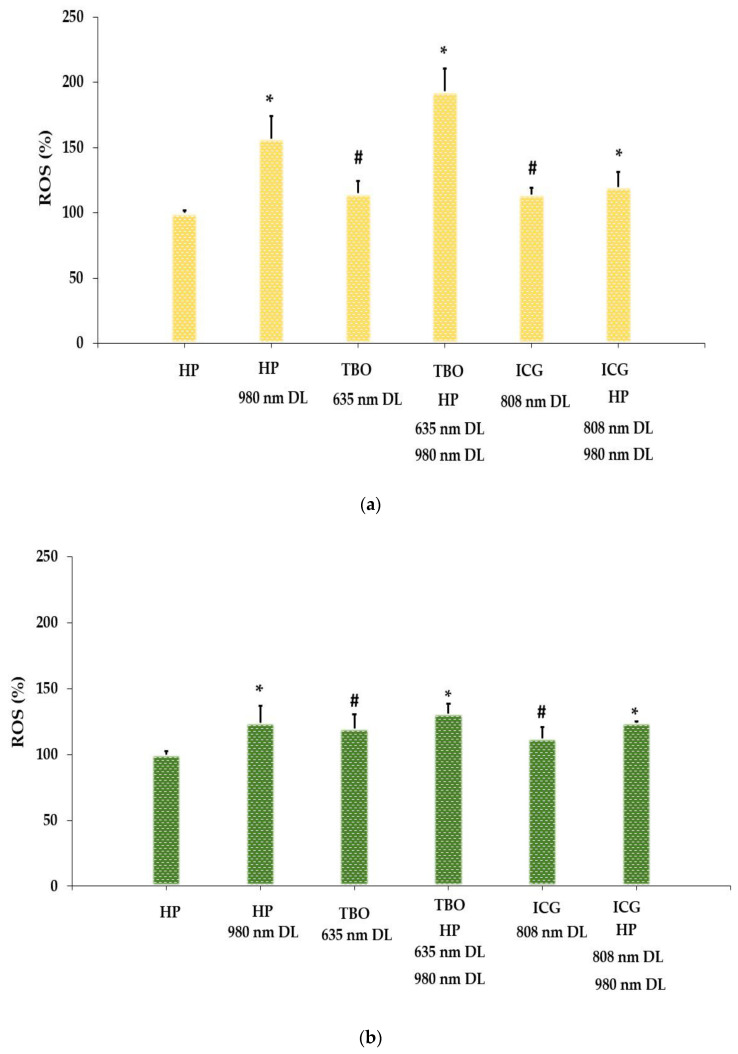

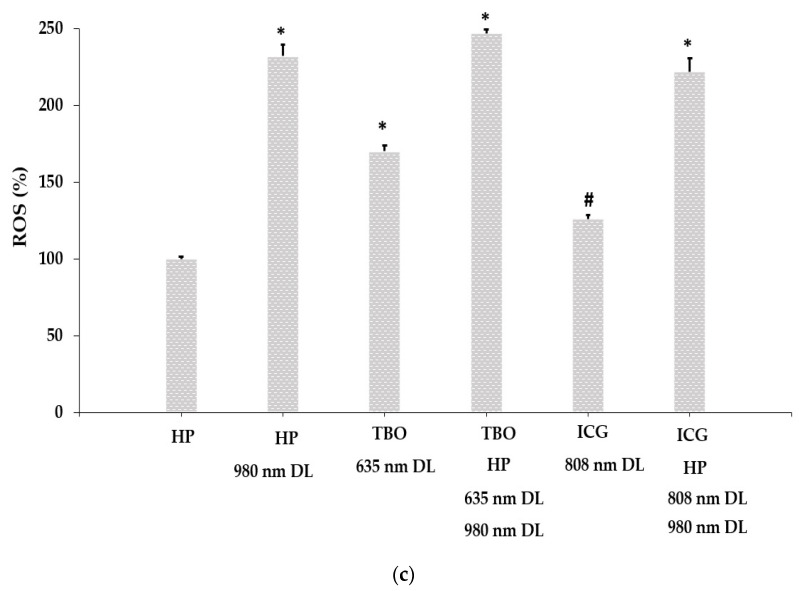
Intracellular reactive oxygen species (ROS) generation in *Staphylococcus aureus* (**a**), *Escherichia coli* (**b**), and *Candida albicans* (**c**) suspension following different treatments. # = *p* < 0.05, * = *p* ≤ 0.001. HP: hydrogen peroxide; DL: diode laser; TBO: toluidine blue O; ICG: indocyanine green.

All treatment groups except HP alone led to an increase in ROS levels compared to the control (Figure 3a–c). Maximum ROS formation was found in the HP-TBO-PDT group, resulting in 1.93-, 1.31-, and 2.47-fold higher ROS generation in *S. aureus*, *E. coli*, and *C. albicans*, respectively, compared to the control cells (*p* < 0.001).

## 4. Discussion

Although the therapeutic potential of light-based treatments continues to increase, the lack of efficient dosimetry and appropriate illumination devices, together with inadequately defined treatment parameters, has also diminished the success of PDT [25]. Furthermore, each photosensitizer has its own advantages and disadvantages [26]. ICG and TBO as photosensitizers, along with exciting light, have been used against periodontal biofilms [27,28,29]. ICG has been approved by the US Food and Drug Administration as a fluorescent agent [30]. ICG acts relatively poorly as a singlet oxygen supplier but has a temperature-raising property and antibacterial ability within biofilm and deep periodontal pockets [31]. In addition, it has also shown low stability in aqueous solutions and its negative charge can lead to a weak interaction with the surface of bacterial cells [32,33]. TBO is an FDA-approved cationic photosensitizer with low excitation energy and high cell membrane permeability [34]. However, the clinical application of TBO is limited, mainly due to the aggregation-induced quenching problem [35]. Alternatively, the fluidic nature of photosensitizers makes retention periods a challenge, which may affect the desired therapeutic outcome [36]. Likewise, the concentration of photosensitizer used to destroy the complex structures of biofilms and yeasts should be higher due to the large size of the cell and the more complex cell structure [37]. On the other hand, the effectiveness of PDT is also influenced by the infection site. Due to the hypo-oxygenic nature of the subgingival milieu, sufficient molecular oxygen is not provided for the production of singlet oxygen [38]. Moreover, the infection-associated biofilms become less oxygenated during their maturation due to oxygen consumption by facultative anaerobes, concentration gradients within the biofilms, and the presence of an extracellular polymeric substance (EPS) [39]. Hence, the reinforced PDT can be considered [36].

In the current study, a reduction of bacteria was observed with all decontamination regimens. The simultaneous administration of photosensitizer with HP followed by dual-wavelength laser irradiation was the most effective protocol, reducing the number of adherent bacteria by approximately 2–3 lg compared to either treatment group alone. The application of PDT to microorganisms alone may cause minor disruption of the membrane. This could increase the exposure of the internal components of the bacteria to HP, which should increase bacterial killing by facilitating access to the interior of the bacteria [40]. HP-based disinfectants have a high affinity for bacteria and their EPS. In addition, they ensure the formation of active oxygen foam, which also has mechanical cleaning properties, and allow deeper penetration of the photosensitizer by reducing the depth of the biofilm [41,42]. The effect of HP on the biofilm mass on the implant surface has been demonstrated [43]. It may be concluded that the exposure of microorganisms to HP leads to an increased uptake of the photosensitizer within the cells, resulting in an increased production of ROS and consequently higher microbial death [40]. Furthermore, photochemical enhancement of the dye occurs through a chemical reaction between HP and ROS produced during PDT, damaging the microbial cell and allowing better penetration of other treatments [40].

With regard to *S. aureus*, Cai et al. investigated the efficacy of combined HP and PDT treatment on the biofilm formed on titanium discs. They concluded that the combined use of antiseptics with TBO-PDT may be a more efficient method for bacterial disinfection of titanium discs compared to treatment alone [42]. HP has previously been shown to be effective against *C. albicans* [44]. This study confirmed previous results showing that the anti-biofilm ratio of HP could be further improved by using a 980 nm diode laser, resulting in 2.5 times more destruction of *Enterococcus faecalis* biofilm [21].

The use of dual-wavelength provides additional benefits in PDT. Nikinmaa et al. investigated *Streptococcus mutans* biofilm susceptibility to irradiation with 810 nm, 405 nm blue light, or dual-wavelength LED light (simultaneously 405 and 810 nm) plus ICG and found a significant difference between dual-wavelength PDT with blue light (405 nm) or 810 nm light alone [32]. De Angelis et al. found that 635 nm diode lasers improved clinical periodontal outcomes when combined with 980 nm diode laser therapy as a dual-wavelength approach [45]. The study by Zhang et al. showed that the silica-coated gold nanorods@curcumin group was irradiated under 405 and 808 nm dual lasers and had a higher antibacterial effect against both *S. aureus* and *E. coli* than that of curcumin or PDT alone [46]. Similarly, Wen et al. developed a nitrogen-doped carbon dots/curcumin nanocomposite and showed good antibacterial properties against *S. aureus* and *E. coli* under dual light source (405 + 808 nm) irradiation [47]. Leanse et al. found that irradiation at the two wavelengths significantly enhanced antimicrobial activity against methicillin-resistant *S. aureus*. A possible explanation for this could be the presence of the EPS produced during biofilm formation, which may have reduced light emission and led to higher radiation exposure to induce antimicrobial effects. Alternatively, the cells within the EPS are metabolically less active, which would explain the higher light dose needed to elicit infinite microbial inhibition [48].

In a variety of stressors, the lethal effect is partly due to the stimulation of ROS accumulation [49]. Following light irradiation, ROS generated by a type I photochemical mechanism lead to membrane degradation, metabolic hydroxylation, and oxidative DNA damage, killing the target cells [50]. Atsumi et al. reported that the production of ROS is both dose and time dependent [51]. In this study, the results show that the combination of HP with photosensitizers can increase ROS production up to 2.5 times after dual-wavelength laser irradiation. However, *E. coli* was less affected compared to other microorganisms, which can be explained by the fact that Gram-negative bacteria are generally resistant to the photosensitization process [52,53]. The transport of molecules through the cell wall of Gram-negative bacteria is regulated in the outer membrane, which is rich in LPS molecules [53].

Also, the results of this study show that TBO-PDT alone or in combination with HP has a better antimicrobial effect than ICG. This could be due to the fact that TBO has a high solubility in the membrane and can therefore easily interact with the bacterial membrane. In addition, cationic dyes can interact better with LPS in Gram-negative bacteria [54].

This study had several limitations. In particular, the results of co-irradiation cannot be compared with previous studies, as this is the first study to demonstrate a possible synergistic antimicrobial effect of two wavelengths and HP on titanium-adherent biofilms of *S. aureus*, *E. coli*, and *C. albicans*. This study has further limitations: Only three bacteria were examined. Furthermore, this study provides data based on a laboratory setting and may not be extrapolated to a real clinical situation. Finally, no comparison was made with other treatment options. Further studies on the other bacterial species, microscopic images, and gene expression systems are required to obtain a clear concept of the mechanisms involved. In addition, cytotoxicity experiments on mammalian cells should be performed.

## 5. Conclusions

The ability of *S. aureus*, *E. coli*, and *C. albicans* to cope with ICG- or TBO-PDT was effectively reduced by the simultaneous irradiation of a 980 nm diode laser and a 635 or 808 nm diode laser. The antibacterial effect of ICG- or TBO-PDT was significantly lower than that of HP-ICG-PDT or HP-TBO-PDT when similar light doses were compared, but the HP-980 nm diode laser improved the antibacterial effect of PDT. This work demonstrates that improving the PDT mechanism by increasing ROS production can enhance photokilling. Optimizing PDT can increase its efficacy and create a new trend for combination therapies against microorganisms. For photosensitizers with lower antimicrobial activity, the protocol can be used to effectively inactivate bacteria.

## Figures and Tables

**Table 1:** Measures of central dispersion for the number of colonies of microorganisms in the study groups.

*Staphylococcus aureus*	Mean (lg CFU/mL)	Std. Deviation	Sig. *	Lower Bound	Upper Bound
**Treatments**					
Control	5.86	0.11			
HP	5.23	0.03	<0.001	0.45	0.92
HP-PDT	4.46	0.13	<0.001	0.94	1.83
TBO-PDT	4.56	0.32	<0.001	0.84	1.73
HP-TBO-PDT	3.96	0.22	<0.001	1.41	2.37
ICG-PDT	4.97	0.21	<0.001	0.40	1.36
HP-ICG-PDT	4.33	0.11	<0.001	1.08	1.97
***Escherichia coli***					
**Treatments**					
Control	6.65	0.05			
HP	6.06	0.03	<0.001	0.38	0.86
HP-PDT	5.26	0.10	<0.001	1.18	1.60
TBO-PDT	5.55	0.05	<0.001	0.89	1.29
HP-TBO-PDT	4.98	0.08	<0.001	1.45	1.90
ICG-PDT	5.67	0.06	<0.001	0.41	0.87
HP-ICG-PDT	5.21	0.12	<0.001	1.23	1.65
***Candida albicans***					
**Treatments**					
Control	6.40	0.46			
HP	6.01	0.13	0.019	0.07	0.70
HP-PDT	4.55	0.21	<0.001	1.53	2.39
TBO-PDT	5.04	0.12	0.001	0.59	2.12
HP-TBO-PDT	3.95	0.22	<0.001	1.69	3.22
ICG-PDT	5.50	0.26	0.011	0.19	1.61
HP-ICG-PDT	4.34	0.31	<0.001	1.29	2.83

HP: hydrogen peroxide; PDT: antimicrobial photodynamic therapy; TBO: toluidine blue O; ICG: indocyanine green; *: significant difference compared with control group.

## Data Availability

Data are contained within the article.
